# Paraesophageal hernia repair is associated with similar safety and efficacy results in octogenarians as compared to younger patients

**DOI:** 10.1007/s00464-026-12944-w

**Published:** 2026-06-08

**Authors:** Kayvan Barekatain, Emily F. Simon, Joshua L. Lyons, Natalie N. Chakraborty, Christina S. Boutros, Trisha Lal, Jill Knepprath, Ayesha Siddiq, Hamza N. Chatha, Saher-Zahra S. Khan, Patrick M. Wieland, Sami O. Abul-Khoudoud, Nicolette M. Winder, Samuel J. Zolin, Jeffrey Marks

**Affiliations:** Department of Surgery, University Hospitals Cleveland Medical Center, 11100 Euclid Ave, Cleveland, OH 44106 USA

**Keywords:** Paraesophageal hernia, Hiatal hernia repair, Octogenarians, Minimally invasive surgery, Surgical outcomes, Recurrence

## Abstract

**Background:**

National database studies suggest elective paraesophageal hernia (PEH) repair in octogenarians is safe but provide limited detail on operative strategy and long-term symptom outcomes. We evaluated perioperative outcomes, operative strategy, and post-discharge functional outcomes in octogenarians undergoing PEH repair compared with younger patients in a contemporary institutional cohort.

**Methods:**

A retrospective review of 2,517 PEH repairs performed at a tertiary academic center from 2010–2023 was conducted. Patients were stratified by age (18–79 vs. ≥ 80 years). Demographics, operative characteristics, 30-day complications, recurrence, reoperation, and postoperative symptoms were compared. Multivariable logistic regression identified predictors of composite adverse outcome (CAO), defined as postoperative GERD, dysphagia, recurrence, or reoperation.

**Results:**

Of 2,517 patients, 195 (7.7%) were ≥ 80 years. Compared with younger patients, octogenarians had lower BMI (26.9 vs. 32.5 kg/m^2^, *p* < 0.001), higher rates of cardiovascular disease (83.6% vs. 61.0%, *p* < 0.001), and more frequent ASA ≥ III (93.2% vs. 69.6% *p* < 0.001). Non-elective surgery was more common in octogenarians (30.6% vs. 6.0%, *p* < 0.001). Surgeons performed gastropexy more than twice as often in octogenarians (43.6% vs. 17.3%, *p* < 0.001) and used mesh reinforcement at higher rates (21.5% vs. 13.0%, *p* = 0.004), while fundoplication rates were similar (33.3% vs. 38.7%, *p* = 0.12). Thirty-day complications were higher in octogenarians (15.9% vs. 9.1%, *p* = 0.006), with no significant difference in mortality (1.0% vs. 0.3%, *p* = 0.15). Hernia recurrence, postoperative GERD, dysphagia, and reoperation were similar between groups (*p* > 0.05 for all). Age ≥ 80 was not independently associated with CAO (OR 1.05, 95% CI 0.72–1.54, *p* = 0.79).

**Conclusions:**

PEH repair in octogenarians is associated with increased perioperative morbidity but similar PEH-related outcomes compared with younger patients. These findings support consideration of elective repair in appropriately selected symptomatic octogenarians and individualized risk assessment based on comorbidities rather than age alone.

Paraesophageal hernias (PEH) occur predominantly in elderly patients, with a median age of presentation between 65 and 75 years [1]. As the population ages, surgeons increasingly encounter octogenarians with symptomatic PEH who may benefit from surgical intervention. However, the optimal management of PEH in this age group remains controversial. While some contemporary evidence supports the safety of PEH repair in carefully selected elderly patients, both advanced age and frailty have been identified as independent predictors of perioperative morbidity, creating ongoing challenges in patient selection and surgical decision-making [2, 3].

The approach to PEH management in elderly patients has evolved considerably. Two decades ago, watchful waiting was recommended for asymptomatic or minimally symptomatic PEH, particularly in patients over 65 years, based on concerns that elective repair mortality outweighed the risks of observation [4]. Advances in minimally invasive techniques and perioperative care have since substantially reduced operative mortality. Updated decision analyses now demonstrate that elective repair leads to increased life expectancy over watchful waiting, even in asymptomatic patients [5]. This reversal of prior recommendations has shifted the management paradigm across all age groups, including octogenarians. National database studies confirm that 30-day mortality following elective PEH repair in patients over 80 years is well below the thresholds that previously justified conservative management [3]. However, most existing literature relies on national databases that capture only 30-day events, with limited data on hernia recurrence, postoperative dysphagia, gastroesophageal reflux disease (GERD), and reoperation in this population.

The objective of this study was to compare perioperative and post-discharge outcomes of PEH repair between octogenarians and younger adults at a high-volume tertiary center. We hypothesized that while octogenarians would experience higher perioperative morbidity, PEH-related outcomes including recurrence, GERD, dysphagia, and reoperation rates would be comparable between age groups.

## Materials and methods

### Study design and patient population

This study was conducted as a retrospective cohort study of patients who underwent paraesophageal hernia (PEH) repair within a tertiary academic health care system between 2010 and 2023. Both abdominal and thoracic approaches were included. Surgeries were conducted by forty-two surgeons of various specialties. This study was approved by the University Hospitals Institutional Review Board (STUDY20190535). All patients who underwent PEH repair during the study period were identified. Patients younger than 18 years of age or those with missing age information were excluded. The remaining cohort was stratified into two groups based on age at the time of surgery: patients aged 18–79 years and those older than 80 years (octogenarians).

### Data collection

Demographic characteristics, comorbidities, operative variables, and postoperative outcomes were collected through retrospective review of the electronic medical record by research fellows. Demographic variables included body mass index (BMI), as well as baseline comorbidities including diabetes mellitus, smoking status, cardiovascular disease, pulmonary disease, and prior abdominal surgery. Operative variables included operative approach, use of mesh reinforcement, fundoplication and fundoplication type, gastropexy, operative time, and timing of surgery (elective, urgent, emergent, or semi-elective). Timing of surgery was defined by clinical presentation and admission status: elective cases were scheduled outpatient procedures; emergent cases required immediate intervention; urgent cases were non-emergent inpatient operations (e.g., for obstruction); and semi-elective cases were performed during the same admission without indications for urgent intervention. Procedural details varied based on surgeon preference.

### Outcomes

The primary outcome of the study was a composite adverse outcome (CAO), defined as the occurrence of postoperative gastroesophageal reflux disease (GERD), dysphagia, hernia recurrence, or reoperation. Secondary outcomes included postoperative GERD, dysphagia, hernia recurrence, reoperation for recurrent hernia, 30-day readmission, and individual postoperative complications including superficial infection, deep infection, organ space infection, leak or perforation, urinary tract infection (UTI), pneumonia, myocardial infarction (MI), pulmonary embolism (PE), and mortality. Postoperative GERD was defined as any persistent gastroesophageal reflux greater than 30 days. Postoperative dysphagia was defined as any persistent postoperative dysphagia greater than thirty days that was related to the procedure. Thirty-day complications were defined as events occurring within 30 days of the index operation. Hernia recurrence was defined as radiographic, endoscopic, or operative evidence of recurrent paraesophageal hernia. Follow-up was defined as the time from the index operation to the most recent documented clinical encounter within the health system.

### Statistical analysis

Statistical analyses were performed using StataSE v 17.0 (StataCorp, College Station, TX). Continuous variables were reported as median with interquartile range (IQR) and compared using the Wilcoxon rank-sum test. Categorical variables were reported as counts with percentages and compared using the chi-square test.

Multivariable logistic regression analysis was performed to identify predictors of composite adverse outcome. Covariates included age group, ASA classification, surgery timing, diabetes mellitus, cardiovascular disease, pulmonary disease, and prior abdominal surgery. For variables with missing data, analyses were performed using available cases. The proportion of missing data for each variable is reported in the tables. Odds ratios with 95% confidence intervals were reported. Multicollinearity was evaluated using variance inflation factors, and model discrimination was assessed using the area under the receiver operating characteristic curve. A two-sided p-value less than 0.05 was considered statistically significant. Additional regression analyses were performed for individual components of the composite outcome when appropriate.

## Results

### Patient cohort

A total of 2,578 patients underwent paraesophageal hernia repair during the study period. After excluding 61 patients younger than 18 years of age or with missing age information, the final analytic cohort consisted of 2,517 adult patients, including 2,322 (92.3%) patients aged 18–79 years and 195 (7.7%) patients aged 80 years or older (octogenarians).

### Baseline characteristics

Baseline patient characteristics are summarized in Table 1. Patients aged 18–79 years had a higher BMI compared with octogenarians (median 32.5 [27.8–39.3] vs. 26.9 [IQR 23.8–29.7], *p* < 0.001) and were more likely to be active smokers (7.4% vs. 2.1%, *p* = 0.015). Cardiovascular disease was present in 61.0% of patients aged 18–79 years compared with 83.6% of octogenarians (*p* < 0.001). Distribution of ASA classification also differed significantly between groups (*p* < 0.001), with octogenarians more frequently classified as ASA III or higher. The younger cohort (18–79) was more likely to have pulmonary disease (38.2% vs. 26.2%, *p* < 0.001). Rates of diabetes mellitus and prior abdominal surgery were similar between groups.

**Table 1:** Baseline patient characteristics

	Age 18–79 *N* = 2,322	Age ≥ 80 *N* = 195	*p* value
*Demographics*			
BMI	32.5 (27.8–39.3)	26.9 (23.8–29.7)	< 0.001
Diabetes mellitus			
No	1,681 (72.4%)	144 (73.8%)	0.90
Yes	309 (13.3%)	25 (12.8%)	
Unknown	332 (14.3%)	26 (13.3%)	
Smoking status			
No	1,829 (78.8%)	166 (85.1%)	0.015
Yes	172 (7.4%)	4 (2.1%)	
Unknown	321 (13.8%)	25 (12.8%)	
Cardiovascular disease			
No	594 (25.6%)	11 (5.6%)	< 0.001
Yes	1,417 (61.0%)	163 (83.6%)	
Unknown	311 (13.4%)	21 (10.8%)	
Pulmonary disease			
No	1,085 (46.7%)	122 (62.6%)	< 0.001
Yes	888 (38.2%)	51 (26.2%)	
Unknown	349 (15.0%)	22 (11.3%)	
Prior abdominal surgery			
No	611 (26.3%)	61 (31.3%)	0.32
Yes	1,406 (60.6%)	111 (56.9%)	
Unknown	305 (13.1%)	23 (11.8%)	
ASA classification			
ASA I	5 (0.3%)	0 (0.0%)	< 0.001
ASA II	524 (30.1%)	10 (6.8%)	
ASA III	1,157 (66.5%)	119 (80.4%)	
ASA IV	27 (1.6%)	10 (6.8%)	
ASA V	3 (0.2%)	0 (0.0%)	
ASA unclassified	23 (1.3%)	9 (6.1%)	

### Operative characteristics

Operative characteristics are shown in Table 2. The majority of procedures in both groups were performed using a minimally invasive approach, most commonly laparoscopy (76.1% in patients aged 18–79 vs. 77.4% in octogenarians, *p* < 0.001). Mesh reinforcement was used in 13.0% of patients aged 18–79 years compared with 21.5% of octogenarians (*p* = 0.004). Fundoplication was performed in 38.7% of patients aged 18–79 years and 33.3% of octogenarians (*p* = 0.12), although distribution of fundoplication type differed between groups (*p* = 0.021). Gastropexy was performed more frequently in octogenarians (17.3% vs. 43.6%, *p* < 0.001). Non-elective surgery was also more common among octogenarians, with urgent, emergent, or semi-elective procedures occurring in 6.0% of patients aged 18–79 years compared with 30.6% of octogenarians (*p* < 0.001). Operative time greater than two hours occurred in 66.8% of patients aged 18–79 years and 75.4% of octogenarians (*p* = 0.014).

**Table 2:** Operative characteristics

	Age 18–79 *N* = 2,322	Age ≥ 80 *N* = 195	*p* value
*Operative characteristic*			
Operative approach			
Laparoscopic	1,766 (76.1%)	151 (77.4%)	< 0.001
Thoracoscopic	6 (0.3%)	2 (1.0%)	
Robotic	183 (7.9%)	10 (5.1%)	
Open	24 (1.1%)	7 (3.6%)	
Unknown	343 (14.8%)	25 (12.8%)	
Mesh Use			
No	1,657 (71.4%)	126 (64.6%)	0.004
Yes	302 (13.0%)	42 (21.5%)	
Unknown	363 (15.6%)	27 (13.8%)	
Fundoplication performed			
No	1,059 (45.6%)	104 (53.3%)	0.12
Yes	899 (38.7%)	65 (33.3%)	
Unknown	364 (15.7%)	26 (13.3%)	
Fundoplication type			
Nissen	546 (60.9%)	36 (55.4%)	0.021
Toupet	283 (31.6%)	18 (27.7%)	
Dor	60 (6.7%)	11 (16.9%)	
Unknown	7 (0.8%)	0 (0.0%)	
Gastropexy			
No	1,558 (67.1%)	85 (43.6%)	< 0.001
Yes	401 (17.3%)	85 (43.6%)	
Unknown	363 (15.6%)	25 (12.8%)	
Surgery timing			
Elective	1,896 (94.0%)	120 (69.4%)	< 0.001
Urgent	71 (3.5%)	33 (19.1%)	
Emergent	22 (1.1%)	9 (5.2%)	
Semi-elective	27 (1.3%)	11 (6.4%)	
Operative time			
< 2 h	771 (33.2%)	48 (24.6%)	0.014
> 2 h	1,551 (66.8%)	147 (75.4%)	
Intraoperative complication			
Bleeding	13 (21%)	0 (0%)	0.21
Leak or perforation	24 (39%)	5 (42%)	
Viscus injury	9 (15%)	4 (33%)	
Other	16 (26%)	3 (25%)	

### Short-term postoperative outcomes

Postoperative outcomes are summarized in Table 3. Thirty-day complications occurred in 9.1% of patients aged 18–79 years compared with 15.9% of octogenarians (*p* = 0.006). Pneumonia occurred in 0.5% of patients aged 18–79 years and 2.1% of octogenarians (*p* = 0.006). Thirty-day readmission occurred in 4.6% of patients aged 18–79 years compared with 8.7% of octogenarians (*p* = 0.010). Thirty-day mortality was low in both groups (0.3% vs. 1.0%, *p* = 0.15).

**Table 3:** Postoperative outcomes

	Age 18–79 *N* = 2,322	Age ≥ 80 *N* = 195	*p* value
*30-day outcome*			
30-day complication			
No	1,786 (76.9%)	143 (73.3%)	0.006
Yes	212 (9.1%)	31 (15.9%)	
Unknown	324 (14.0%)	21 (10.8%)	
Complication type			
Superficial infection	3 (0.1%)	0 (0.0%)	0.62
Deep infection	2 (0.1%)	0 (0.0%)	0.68
Organ space infection	5 (0.2%)	1 (0.5%)	0.41
Leak or perforation	11 (0.5%)	2 (1.0%)	0.30
Urinary tract infection	8 (0.3%)	1 (0.5%)	0.71
Pneumonia	11 (0.5%)	4 (2.1%)	0.006
Myocardial infarction	2 (0.1%)	0 (0.0%)	0.68
Pulmonary embolism	4 (0.2%)	0 (0.0%)	0.56
Readmission < 30 days	106 (4.6%)	17 (8.7%)	0.010
Death	8 (0.3%)	2 (1.0%)	0.15
Other	86 (3.7%)	13 (6.7%)	0.041
*PEH related outcomes*			
Recurrence			
No	1,526 (65.7%)	130 (66.7%)	0.41
Yes	472 (20.3%)	44 (22.6%)	
Unknown	324 (14.0%)	21 (10.8%)	
Postoperative GERD			
No	1,655 (71.3%)	149 (76.4%)	0.29
Yes	340 (14.6%)	25 (12.8%)	
Unknown	327 (14.1%)	21 (10.8%)	
Postoperative dysphagia			
No	1,711 (73.7%)	145 (74.4%)	0.42
Yes	283 (12.2%)	28 (14.4%)	
Unknown	328 (14.1%)	22 (11.3%)	
Reoperation			
No	1,887 (81.3%)	167 (85.6%)	0.32
Yes	112 (4.8%)	7 (3.6%)	
Unknown	323 (13.9%)	21 (10.8%)	
Composite adverse outcome (CAO)			
No	1,650 (71.1%)	132 (67.7%)	0.32
Yes	672 (28.9%)	63 (32.3%)	

### PEH-related outcomes

PEH-related outcomes were similar between groups. Hernia recurrence occurred in 20.3% of patients aged 18–79 years and 22.6% of octogenarians (*p* = 0.41). Postoperative GERD was reported in 14.6% of patients aged 18–79 compared with 12.8% of octogenarians (*p* = 0.29). Postoperative dysphagia occurred in 12.2% and 14.4%, respectively (*p* = 0.42). Reoperation for recurrent hernia occurred in 4.8% of patients aged 18–79 years compared with 3.6% of octogenarians (*p* = 0.32). The overall rate of composite adverse outcome was 28.9% in patients aged 18–79 years and 32.3% in octogenarians (*p* = 0.32).

### Multivariable analysis

Multivariable logistic regression evaluating predictors of composite adverse outcome is shown in Table 4. Age greater than 80 years was not independently associated with composite adverse outcome (OR 1.05, 95% CI 0.72–1.54, *p* = 0.79). Prior abdominal surgery was independently associated with increased odds of composite adverse outcome (OR 1.38, 95% CI 1.11–1.72, *p* = 0.004). ASA classification, surgery timing, diabetes mellitus, and pulmonary disease were not significantly associated with composite adverse outcome. Model diagnostics demonstrated no significant multicollinearity (mean VIF 2.03), and model discrimination was modest with an area under the ROC curve of 0.56.

**Table 4:** Multivariable logistic regression for composite adverse outcome

	Odds ratio	*p* value	Confidence interval
Age			
18–79 years	Reference		
≥ 80 years	1.05	0.79	0.72–1.54
ASA classification			
ASA I	Reference		
ASA II	0.65	0.64	0.11–3.93
ASA III	0.69	0.69	0.11–4.22
ASA IV	0.88	0.90	0.13–6.10
ASA V	0.57	0.72	0.03–11.56
ASA unclassified	0.75	0.78	0.10–5.56
Surgery timing			
Elective	Reference		
Urgent	0.84	0.49	0.51–1.38
Emergent	0.92	0.85	0.38–2.20
Semi-elective	0.92	0.82	0.43–1.94
Diabetes mellitus			
No	Reference		
Yes	0.92	0.55	0.69–1.22
Cardiovascular disease			
No	Reference		
Yes	1.22	0.10	0.97–1.53
Pulmonary disease			
No	Reference		
Yes	0.95	0.61	0.77–1.16
Previous abdominal surgery			
No	Reference		
Yes	1.38	0.004	1.11–1.72

To further assess the individual components of the composite endpoint, separate multivariable logistic regression models were performed for postoperative GERD, postoperative dysphagia, hernia recurrence, and reoperation. In these analyses, age greater than 80 years was not independently associated with postoperative GERD (OR 0.68, 95% CI 0.40–1.16, *p* = 0.16), postoperative dysphagia (OR 1.18, 95% CI 0.73–1.92, *p* = 0.50), hernia recurrence (OR 0.93, 95% CI 0.61–1.41, *p* = 0.74), or reoperation (OR 0.63, 95% CI 0.26–1.52, *p* = 0.30). In the recurrence model, cardiovascular disease (OR 1.29, 95% CI 1.00–1.67, *p* = 0.048) and prior abdominal surgery (OR 1.35, 95% CI 1.05–1.72, *p* = 0.017) were independently associated with recurrence. No other covariates were consistently associated with the individual secondary outcomes.

## Discussion

In this large single-institution cohort of 2,517 patients undergoing paraesophageal hernia repair, octogenarians experienced higher rates of 30-day complications (15.9% vs. 9.1%) and readmission (8.7% vs. 4.6%) compared with younger patients. However, age greater than 80 years was not independently associated with hernia recurrence, postoperative GERD, dysphagia, reoperation, or a composite adverse outcome of these. These findings suggest that while octogenarians face increased perioperative morbidity, post-discharge functional outcomes are comparable to younger patients.

These results have important implications for surgical decision-making in elderly patients with symptomatic paraesophageal hernia. The historical paradigm of watchful waiting in patients over 65 years was based on decision analyses suggesting that elective repair mortality exceeded the risks of observation [4]. Contemporary data, including the present study, demonstrate that this threshold has been surpassed, 30-day mortality in our octogenarian cohort was 1.0%, consistent with national database estimates of 1.3–1.8% for elective repair in this age group [3, 6]. More importantly, our data extend beyond perioperative outcomes to demonstrate that the benefits of repair, symptom relief and hernia control, are durable in octogenarians.

The higher rate of non-elective surgery among octogenarians (30.6% vs. 6.0%) is consistent with prior reports demonstrating that elderly patients are disproportionately likely to present emergently [7]. Emergency repair carries substantially higher mortality than elective intervention, and prior studies show that emergency presentation rather than age itself is a primary driver of mortality in this population [1, 6]. This finding raises the possibility that a subset of elderly patients may not be referred for or offered elective repair until disease progression results in urgent or emergent presentation. Although our dataset does not capture prior outpatient evaluation, this pattern highlights a potential opportunity for earlier identification and risk stratification. These findings, combined with our data showing acceptable elective outcomes, support the importance of timely elective evaluation and consideration of repair in appropriately selected candidates to potentially prevent catastrophic acute complications.

The increased 30-day complication rate in octogenarians was driven primarily by pneumonia (2.1% vs. 0.5%). This aligns with existing literature demonstrating that while octogenarians experience higher general medical complications and longer hospital stays, intraoperative and postoperative surgical complication rates are comparable to younger patients [8]. Our findings support the concept that advanced age confers medical rather than surgical risk, and that careful perioperative optimization, particularly pulmonary rehabilitation and aspiration precautions, may mitigate some of these complications.

The comparable recurrence rates between octogenarians and younger patients (22.6% vs. 20.3%, *p* = 0.41) are notable given the differences in operative strategy and hernia complexity. Surgeons adapted their approach in octogenarians, performing gastropexy more than twice as frequently (43.6% vs. 17.3%, p < 0.001) and using mesh reinforcement at higher rates (21.5% vs. 13.0%, *p* = 0.004). Recent randomized trial data have demonstrated that anterior gastropexy reduced 1-year recurrence by approximately 21% [9]. The higher gastropexy rate in our octogenarian cohort may have contributed to the equivalent recurrence rates observed despite the more complex hernia anatomy in this group. Fundoplication rates were similar between groups (33.3% vs. 38.7%, *p* = 0.12), suggesting that surgeons did not systematically avoid anti-reflux procedures in elderly patients, which may explain the comparable postoperative GERD rates (12.8% vs. 14.6%, *p* = 0.29).

Prior abdominal surgery emerged as the only independent predictor of composite adverse outcome (OR 1.38, 95% CI: 1.11–1.72, *p* = 0.004) and was also associated with hernia recurrence (OR 1.35, 95% CI: 1.05–1.72, *p* = 0.017). This finding is consistent with the technical challenges posed by adhesions and altered anatomy in patients with prior operations. Cardiovascular disease demonstrated a trend toward increased composite adverse outcome (OR 1.22, 95% CI 0.97–1.53, *p* = 0.095), suggesting that comorbidity burden rather than chronological age may be more relevant to outcomes. Notably, ASA classification and surgery timing were not independently associated with adverse outcomes in multivariable analysis, though this may reflect the modest discriminative ability of our model (AUC 0.56).

This study has several limitations. The retrospective design introduces potential selection bias, as octogenarians offered surgery may represent a healthier subset of elderly patients. The single-institution nature limits generalizability, though it ensures consistency in surgical technique and follow-up protocols. Follow-up duration was variable, and we did not have complete data on time to recurrence of symptom assessment. Additionally, missing covariate data reduced the analytic sample for multivariable models to 1,777 patients (70.6%). The definition of recurrence included radiologic findings, which may overestimate clinically significant recurrence. We also were unable to determine whether patients presenting for urgent or emergent repair had been previously evaluated for elective surgery, as longitudinal outpatient data were not consistently available. Our cohort includes only patients who underwent operative repair, limiting our ability to assess the proportion of elderly patients evaluated for paraesophageal hernia who ultimately proceeded to surgery. Additionally, we did not assess frailty using validated instruments, which may be more predictive of outcomes than chronological age alone [2].

Despite these limitations, this study represents one of the largest single-institution analyses of paraesophageal hernia repair in octogenarians with assessment of outcomes beyond the 30-day perioperative window. The findings support elective repair in symptomatic octogenarians, with the understanding that perioperative morbidity is increased but post-discharge outcomes are comparable to younger patients. Future studies should incorporate frailty assessment and prospective quality-of-life measures to further refine patient selection in this growing population.

In conclusion, paraesophageal hernia repair in octogenarians is associated with increased perioperative morbidity but similar rates of recurrence, postoperative GERD, dysphagia, and reoperation compared with younger patients. Age greater than 80 years was not independently associated with composite adverse outcome. These findings support consideration of elective repair in symptomatic elderly patients and highlight the importance of individualized risk–benefit discussions that account for comorbidity burden and surgical urgency rather than chronological age alone.
